# Age-related differences in gait symmetry obtained from kinematic synergies and muscle synergies of lower limbs during childhood

**DOI:** 10.1186/s12938-022-01034-2

**Published:** 2022-09-04

**Authors:** Qiliang Xiong, Jinliang Wan, Shaofeng Jiang, Yuan Liu

**Affiliations:** 1 Key Laboratory of Nondestructive Testing, Ministry of Education, Nanchang Hangkong University, Nanchang, Jiangxi China; 2 Department of Biomedical Engineering, Nanchang Hangkong University, Nanchang, Jiangxi China; 3 Department of Rehabilitation, Children’s Hospital of Chongqing Medical University, Chongqing, China

**Keywords:** Gait symmetry, Kinematic synergy, Muscle synergy, Children

## Abstract

The age-related changes of gait symmetry in healthy children concerning individual joint and muscle activation data have previously been widely studied. Extending beyond individual joints or muscles, identifying age-related changes in the coordination of multiple joints or muscles (i.e., muscle synergies and kinematic synergies) could capture more closely the underlying mechanisms responsible for gait symmetry development. To evaluate the effect of age on the symmetry of the coordination of multiple joints or muscles during childhood, we measured gait symmetry by kinematic and EMG data in 39 healthy children from 2 years old to 14 years old, divided into three equal age groups: preschool children (G1; 2.0–5.9 years), children (G2; 6.0–9.9 years), pubertal children (G3; 10.0–13.9 years). Participants walked barefoot at a self-selected walking speed during three-dimensional gait analysis (3DGA). Kinematic synergies and muscle synergies were extracted with principal component analysis (PCA) and non-negative matrix factorization (NNMF), respectively. The synergies extracted from the left and right sides were compared with each other to obtain a symmetry value. Statistical analysis was performed to examine intergroup differences. The results showed that the effect of age was significant on the symmetry values extracted by kinematic synergies, while older children exhibited higher kinematic synergy symmetry values compared to the younger group. However, no significant age-related changes in symmetry values of muscle synergy were observed. It is suggested that kinematic synergy of lower joints can be asymmetric at the onset of independent walking and showed improving symmetry with increasing age, whereas the age-related effect on the symmetry of muscle synergies was not demonstrated. These data provide an age-related framework and normative dataset to distinguish age-related differences from pathology in children with neuromotor disorders.

## Introduction

Gait symmetry is utilized as an indicator of neurologic function [1]. Evidence of the neurological basis of gait symmetry can be observed in studies evaluating the symmetry of cortical connectivity in both hemispheres of the brain [2]. Another study found that asymmetry is caused by non-lesioned neuronal activity and that only measures of symmetry were associated with global recovery scores [3]. Taken together with clinical observations, there appears to be a link between gait symmetry and the nervous system. Therefore, a better understanding of how the nervous system's symmetrical activity influences gait symmetry in typically developing children may allow the development of treatment programs for children with neurological gait asymmetry. However, to our best knowledge, no research has been conducted to understand the age-related symmetrical activity of the nervous system influencing gait symmetry, and one aspect where this is lacking is the symmetry measures calculated from various gait features including spatial–temporal [4], kinetic [5], kinematic [6] features are insufficient to describe the state of the nervous system.

Motor synergy, also known as primitives, is a hypothesis that can be used to describe the state of the nervous system [7–9]. Researchers have discovered that the neuromotor system coordinates dimensionality in joints, muscles, and neural activity in the last few decades [10, 11]. Specifically, the central nervous system (CNS) simplifies the control of complex motor behaviors by merging a small number of primitives [9, 12, 13], each of that is a functional unit that specifies a multiple-degree-of-freedom coordination pattern. Motor primitives at different levels (commonly referred to as synergies) have been identified in this context, supporting the modular control hypothesis [9, 14]. For example, studies on the neural control of muscles show that a few muscle synergies sufficiently describe various muscle activation patterns in human locomotion [15, 16]. The joint angles of lower limb segments can be successfully explained by a few kinematic synergies in some typical human movements [17–19]. Taken together, the CNS organizes these redundant systems by generating the common motor command to coordinate multiple muscles or joints [20, 21]. This coordinated pattern of muscle activities or joint angle movements can be extracted as common components using dimensionality reduction techniques, such as principal component analysis (PCA) [19, 22] or non-negative matrix factorization (NNMF) [23, 24].

Since gait symmetry is controlled by an individual's CNS, incomplete maturation and aging-related declines in the CNS may influence gait symmetry in children and the elderly, respectively. Gait asymmetry caused by aging and neurological injuries has been extensively studied in the elderly [25–28], and the individual development and improvement of the CNS during childhood should also be considered. Furthermore, given the rapid development and maturation of the CNS during early childhood [29, 30], it is plausible to assume that asymmetry of motor synergies exists with the initiation of the independent walking stage and that this asymmetry tends to develop toward a more symmetrical synergies with age increased.

To test this hypothesis, we compared the gait symmetry extracted from lower extremity kinematic synergies and muscle synergies in children among three different age groups: preschool children (G1; 2.0–5.9 years), children (G2; 6.0–9.9 years), pubertal children (G3;10.0–13.9 years). One way to quantify the kinematic synergy is the principal component analysis (PCA) method [22, 31, 32], which is a statistical approach that uses a principal component (PC) (i.e., kinematic synergy weight) and the corresponding timing coefficients to cluster individual joint motions into functional units [32]. Because negative weights have no physiological significance for muscle activations [23, 24, 33], muscle synergies were extracted with non-negative matrix factorization (NNMF) [34], in which time-invariant synergies with fixed weights among muscles (i.e., muscle synergy weight) were recruited by timing activation coefficients. Then, the synergy weight and timing coefficients of the identified kinematic synergies and muscle synergies in the left and right lower limbs were compared to obtain a similarity value that denoted their symmetry. These values were referred to as the “weight symmetry” (for synergy weight symmetry) and “timing symmetry” (for timing coefficient symmetry), as detailed in the method section.

## Results

### Number of kinematic synergies and muscle synergies

The PCA revealed that a total of 9 PCs of kinematic synergies were generated; the explained variance of each PCs is shown in Table 1. According to the variance threshold (> 90% explained variance) defined by previous studies [35], all the subjects identified three to four kinematic synergies for each side, of which 7 subjects in G1, 8 subjects in G2, and 6 subjects in G3 demonstrated no difference in the number of kinematic synergies between the left and right side, while 6 subjects in G1, 5 subjects in G2, and 7 subjects in G3 identified a left–right difference in the number of kinematic synergies by only one. The averaged number of kinematic synergies across the three groups is shown in Table 2.

**Table 1:** The percentage of the variance explained by each PC of kinematic synergies is shown, values are expressed as the mean ± SD

	Group1 (2.0–5.9 years)		Group 2 (6.0–9.9 years)		Group 3 (10.0–13.9 years)	
	Left	Right	Left	Right	Left	Right
PC1	45.90 ± 6.64	48.66 ± 6.59	49.06 ± 6.26	48.97 ± 7.81	45.79 ± 8.47	45.64 ± 8.34
PC2	26.00 ± 2.87	24.48 ± 4.19	24.33 ± 3.37	23.05 ± 4.02	25.12 ± 4.07	24.66 ± 3.40
PC3	14.88 ± 2.36	13.94 ± 1.86	13.87 ± 2.12	14.13 ± 2.51	15.94 ± 3.31	14.96 ± 3.01
PC4	7.59 ± 2.33	6.97 ± 1.72	7.14 ± 1.70	8.06 ± 1.29	7.40 ± 1.87	7.81 ± 2.79
PC5	3.18 ± 1.01	3.56 ± 1.08	3.27 ± 0.81	3.34 ± 0.79	3.41 ± 0.89	3.88 ± 1.20
PC6	1.40 ± 0.45	1.45 ± 0.44	1.35 ± 0.54	1.40 ± 0.57	1.40 ± 0.58	1.75 ± 0.88
PC7	0.65 ± 0.20	0.58 ± 0.23	0.62 ± 0.24	0.67 ± 0.29	0.63 ± 0.34	0.84 ± 0.47
PC8	0.28 ± 0.10	0.24 ± 0.13	0.24 ± 0.11	0.26 ± 0.09	0.21 ± 0.11	0.33 ± 0.27
PC9	0.08 ± 0.04	0.08 ± 0.03	0.06 ± 0.01	0.07 ± 0.04	0.06 ± 0.04	0.08 ± 0.07

**Table 2:** The number of kinematic synergies and muscle synergies identified in the three age groups (G1, G2, G3)

	Kinematic synergy			Muscle synergy		
	Left	Right	Maximum of left and right	Left	Right	Maximum of left and right
G1	3.69 ± 0.48	3.69 ± 0.48	3.92 ± 0.27	2.30 ± 0.48	2.07 ± 0.27	2.30 ± 0.48
G2	3.46 ± 0.51	3.61 ± 0.50	3.61 ± 0.50	2.15 ± 0.37	2.30 ± 0.48	2.30 ± 0.48
G3	3.38 ± 0.50	3.61 ± 0.65	3.76 ± 0.59	2.23 ± 0.43	2.30 ± 0.48	2.46 ± 0.51

Table 2 also reports the number of muscle synergies that are able to fulfill the VAF threshold (> 90% VAF overall and > 75% VAF per muscle). All the subjects identified two to three muscle synergies, of which 9 subjects in G1, 11 subjects in G2, and 8 subjects in G3 demonstrated no difference in the number of muscle synergies between the left and right side, while 4 subjects in G1, 2 subjects in G2, and 3 subjects in G3 identified a left–right difference in the number of muscle synergies by only one.

### The symmetry of kinematic synergies

**Fig. 1:**
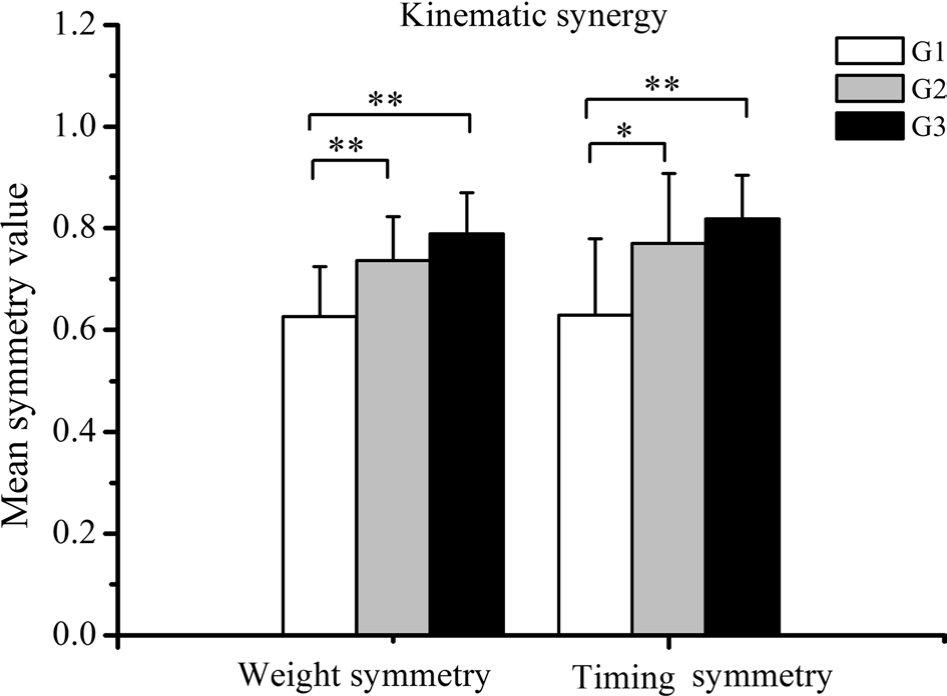
The mean symmetry value of kinematic synergies for the three groups of tested children. Left column: calculation according to the scalar dot product between the weight of kinematic synergies on both sides. Right column: calculation according to the Pearson correlation coefficient between the corresponding timing coefficients of kinematic synergies on both sides

The ANOVA analysis detected a significant effect of age on the mean weight symmetry value of kinematic synergies (*F* = 11.39, *p* < 0.001). Symmetry values tended to increase with children’s age. As shown in Fig. 1, children in G3 exhibited a higher mean weight symmetry value with respect to G1 (*p* < 0.01). Children in G2 were found characterized by a higher mean weight symmetry value when compared with G1 (*p* < 0.01). The results of the statistical analysis performed with the timing symmetry value of kinematic synergy were quite similar. The ANOVA analysis detected a significant main effect of age on the mean timing symmetry value of kinematic synergy (*F* = 7.76, *p* < 0.01). The post hoc analysis found the same differences between the groups as previously described.

**Fig. 2:**
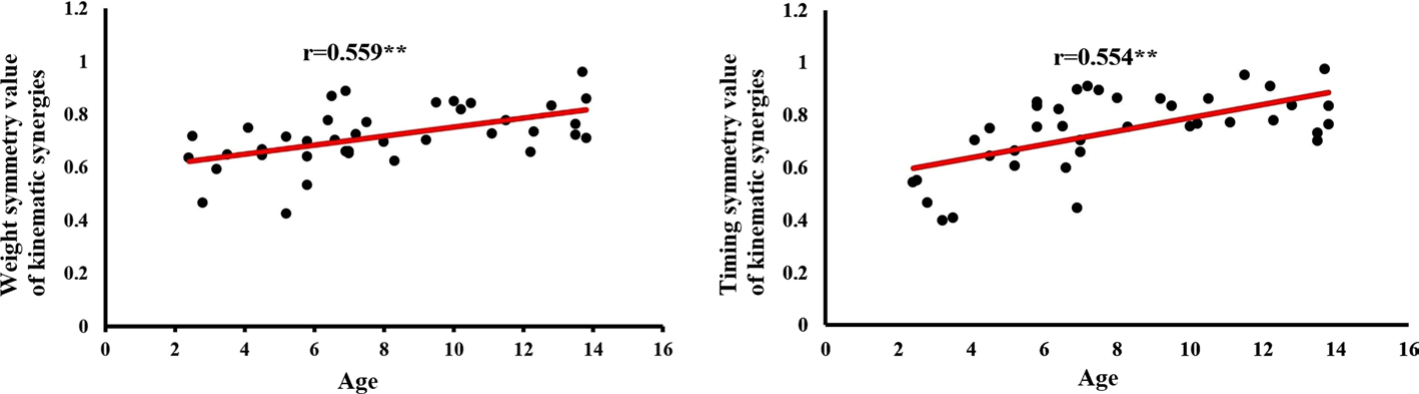
Relationship between age and the mean weight symmetry value (left figure) and the mean timing symmetry value (right figure) of kinematic synergies extracted from both sides

In addition, Fig. 2 shows the trend of symmetry values of kinematic synergy with increasing age. It is possible to observe a positive strong relationship between age and the mean weight symmetry value (*r* = 0.559, *p* < 0.01) and the mean timing symmetry value (*r* = 0.554, *r* < 0.01).

### The symmetry of muscle synergies

No main significant group effect was detected by statistical analysis with regard to the mean weight symmetry value of muscle synergies (*F* = 0.53, *p* = 0.949) or the mean timing symmetry value of muscle synergies (*F* = 1.70, *p* = 0.197).

## Discussion

This study aimed to examine the effect of age on the gait symmetry of the coordination of multiple joints or muscles activity during childhood, symmetry values were obtained from kinematic synergies and muscle synergies of the left and right lower limbs in 39 healthy children from 2 years old to 14 years old, who were separated into three age groups. The findings showed that the effect of age on the symmetry values extracted by kinematic synergies was significant during childhood, with older children having higher kinematic synergy symmetry values compared with younger children. However, no age-related changes in muscle synergy symmetry value were demonstrated. The findings of this study are further explained in the following sections.

### Effect of age on the symmetry of kinematic synergies

The coordinated movement of multiple joints and muscles is necessary for the complex task of walking. It has been assumed that the joints or muscles are engaged in groups with a fixed weight, i.e., the weight of synergies, to overcome the complexity of controlling a large number of degrees of freedom (DoF) [36, 37]. Based on this hypothesis, the CNS generates a large range of physical activities by the flexible recruitment of a limited number of synergies over time, i.e., the timing coefficients of synergies. Extending to the current study, our results of initial kinematic synergy asymmetries in the G1 (2.0–5.9 years) group could be the result of a “suboptimal” motor command, due to the unmatured CNS. It is well established that an appropriate level of coordination among the multiple joints is required during walking. Without the appropriate motor command from the CNS, the high number of joint combinations available during the early stage of independent walking would cause the net output of many joints to be highly variable, resulting in both efficient and inefficient patterns. As age increases, the CNS tends to abandon the inefficient combinations for improving locomotion efficiency development. The motor command of the kinematic synergies of the left and right limbs gradually tends to be the “optimal” and “identical” patterns, and the symmetric performance between the left and right sides improves with age.

However, the main role of the CNS in generating kinematic synergy has been questioned by other authors who suggested that the CNS may not directly control the kinematics (joint angles), but the “joint constraint” [38]. Another hypothesis about the kinematic synergies suggested that the "joint constraint" interferes with the coordinative structure in joint motions required to optimize postural stability [38]. Considering this hypothesis, the initial kinematic synergy asymmetries in the G1 (2.0–5.9 years) group can be attributed to a lack of postural capacity to modulate gravity forces during walking. This has been proved by the previous study to take at least 3–4 years of walking experience [39], inconsistent with the report of Wheelwright et al., who found that normal gait in 3–18 years old children has some degree of asymmetry in terms of temporal and spatial gait metrics [40]. In children aged 4–10 years, Diop et al. observed asymmetrical ground reaction force and temporal–spatial characteristics during their gait [41].

In Fig. 2, it is possible to observe a positive strong relationship (Spearman’s *r* = 0.559, *p* < 0.01 and *r* = 0.658, *r* < 0.01) between the weight and timing symmetry of kinematic synergies and age. But it is hard to draw the conclusion when changes in gait symmetry of kinematic synergies occur and at what age. According to the existing literature, gait development is impacted by age in healthy children [42], specifically, in children aged 7–12, sagittal plane gait kinematics for the ankle, knee, and hip joints were demonstrated to be impacted by age [40]. However, age-related gait maturation has been reported with different conclusions. For example, reaching adult-like patterns of joint dynamics has been reported to occur at 3 years [43], 5 years [44], 7–8 years [45], 9–13 years [46], 10 years [47], and more than 10 years [48]. More recent work suggests normalized step length and gait speed stabilize from 5 to 13 years with little change from the age of 7 [49]. Other research, however, reported that 7-year-old children (*n* = 15) may lack the neuromuscular maturity to generate a mature or adult-like gait pattern, suggesting that it is not fully developed or mature by the age of seven [50].

### Effect of age on the symmetry of muscle synergies

**Fig. 3:**
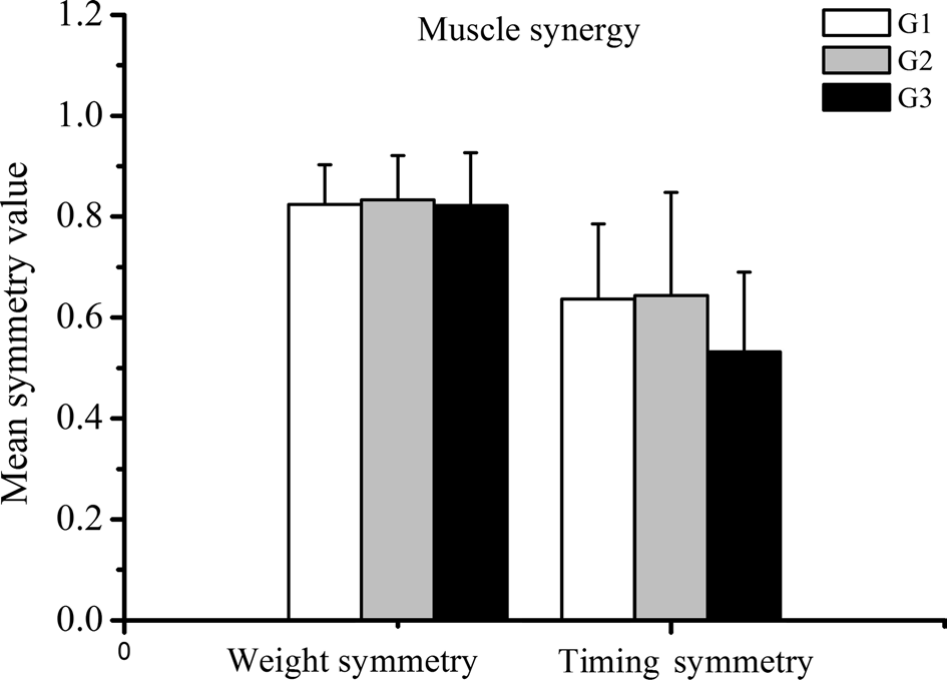
The mean symmetry value of muscle synergies for the three groups of tested children. Left column: calculation according to the scalar dot product between the weight of muscle synergies on both sides. Right column: calculation according to the Pearson correlation coefficient between the corresponding timing coefficients of muscle synergies from both sides

It has been proposed that the activation of muscle synergies is cortically controlled, based on the results from primates [51] and humans [52], the development of cortical control ability is accompanied by increased age during childhood [53]. Therefore, we expected to find improvement of equal characteristics of muscle synergies on both sides. However, this hypothesis about age-related changes in muscle synergy symmetry was not supported, and no difference in muscle synergy symmetry values was found among the age groups (as shown in Fig. 3).

By inspection of Fig. 3, it can be understood that the weight symmetry of muscle synergies showed a good symmetry performance across all three groups (G1: 0.82 ± 0.07; G2:0 .83 ± 0.08; G3:0. 82 ± 0.10), while the timing symmetry of muscle synergies showed poorer symmetry performance across all the groups (G1:0.6 3 ± 0.14; G2: 0.64 ± 0.20; G3: 0.53 ± 0.15). The symmetry of weight and timing coefficient of muscle synergies were both independents of age. Based on the hypothesis of muscle synergy during rhythmic movement (e.g., walking), the basic weight of muscle synergies may be produced by spinal central pattern generators (CPG) [54], which are modulated by sensory feedback pathways within the spinal cord. The corresponding timing coefficient of muscle synergies represents a variety of hierarchical neural pathways for robust motor control, such as descending cortical pathways, and brainstem pathways [55]. The good performance of the weight symmetry of muscle synergies may suggest that CPG, which is located at the spinal cord level, has already developed in new walkers, and thus could account for the absence of age-related effect on the weight symmetry of muscle synergies. Moreover, the absence of group difference in the timing symmetry of muscle synergies may be attributed to the various muscular coordination methods and compensatory patterns of early independent walking during childhood, because Chvatal and Ting noted that muscle synergies may be recruited by different neural circuits for a common motor task [55]. Similarly, a previous study on human walking revealed that several muscle synergies showed regular, phasic regulation during slow walking, but some other muscle synergies were recruited irregularly or not at all [56]. The nature of muscle synergies during the early walking stage might be explained by the preferred control strategies after the acquisition of proficiency in executing this gross motor skill. Furthermore, the neuromuscular system, as Cheung et al. [23] pointed out, is a complex mix of descending and ascending neural circuits that interact with each other, with alterations occurring in subcortical areas as well.

On the other hand, a lot of muscles are involved in human locomotion, making movement complex and smooth. Since the difficulty of measuring locomotion in young kids, we only measured five dominant muscles of the legs. Meanwhile, the variability of muscle timing during gait has been described [57], and slight variations of EMG patterns exist in the normal gait of individuals especially children [58], within-session EMG variability is twice as high in children aged 6–8 years as in adults [59]. As a consequence, quite a limited number of muscles and large muscle activity variability among younger children may hinder the understanding/evaluation of the strategy of how the nervous system organizes muscle synergies. If this is the case, more skeletal muscles need to be included in the synergy analysis.

### The effect of age was only manifested on the symmetry of kinematic synergies, rather than the symmetry of muscle synergies

In the current study, we tried to verify the effect of age on the gait symmetry measures obtained from two levels of motor synergies: kinematic synergies and muscle synergies. Considering the systematic relations between kinematic synergies and muscle synergies, it was postulated that muscle synergies are the source of kinematic synergies [60, 61], and the symmetry of muscle synergies should be able to reveal similar age-related changes as kinematics synergies. But our results demonstrated that the effect of age was only manifested in the kinematic synergies, rather than muscle synergies. This may raise the question: if the age-related improvement of kinematic synergies symmetry is not due to the improved symmetry of the lower extremity muscle synergies, what else factors could have an effect?

According to the conceptual model of motor synergy, the existing muscle synergies give rise to a smaller set of producible forces, which accordingly produces a smaller set of kinematics and, ultimately, an even smaller set of motor behaviors [62]. The hierarchical structure of motor synergies indicates that the kinematic performance can be regarded as the output of muscle synergies [60, 61], but kinematic output performance is not dependent solely on muscle synergies. Combined with our results, in addition to the muscle synergies of the lower extremities, other factors, such as arm movement, can contribute to the improvement of gait symmetry during childhood. While the arms might not have a direct impact on the lower extremity’s symmetry, they could have an important impact on children s stability during walking [63]. It should be noted that the early stages of free walking are characterized by extensive trunk oscillations and a broad base of support, which help to manage instability in new walkers [64]. This instability is a sign of the yet insufficient equilibrium and limited coordination skills. According to a previous study, even older kids up to the age of 10 are less effective at controlling their dynamic balance than adults [65]. Accordingly, the somatosensory system needs to be developed as well, in order to improve gait balance and stability control [5]. Especially, it has been proved that increases in gait stability were suggested to be linked to decreases in gait asymmetry [66]. Taken together, in addition to the muscle synergies of lower extremities, other factors such as upper-limb movement and somatosensory system developments may be responsible for the age-related improvement of kinematic synergies symmetry.

### Limitations

To our knowledge, our study is novel in that the effect of age on the symmetric activity of multiple joints and muscles between the lower limbs has not previously been studied in healthy children. We acknowledge some limitations in this study. First, our findings are based on a cross-sectional investigation, whereas a longitudinal study design could provide additional insight into the development of gait symmetry. Second, the multiple joints and muscles only considered the lower limbs, it is reasonable that arms also significantly move in variable situations during the first years of independent walking and development towards a symmetric movement as well. Future research could look at the symmetry of upper-limb kinematic and muscle synergies. Third, although we were able to replicate previous studies reporting kinematic synergies or muscle synergies analysis during gait, no studies are reporting the effect of age on the symmetry derived by kinematic synergies and muscle synergies, preventing direct comparison with our findings. As a result, higher sample sizes should be used to confirm the findings of this study. Fourth, in the current study, we excluded the first and last steps of each sequence (initiation and termination) to reduce the acceleration or deceleration states of walking, However, some studies reported that healthy adults reach steady-state walking speed with the third step [67, 68]. It seems the 3rd or even the 4th step might be the appropriate onset of steady state during walking. Therefore, the gait initiation might influence the results. At last, the simple normalization approach used during the PCA extraction procedure in our study might magnify the effects of minor joint DoFs against the major ones, and there is a need for more sophisticated normalization procedures that neither ignore nor exaggerate these effects. Even with the limitations listed above, the promise of measuring gait symmetry and the underlying mechanism of motor control that contributes to gait symmetry development in children is exciting. Furthermore, presenting normative values and age-related changes will lead to a better understanding of gait symmetry development, and consequently a better interpretation of neurological gait abnormalities in children.

## Conclusion

In conclusion, kinematic synergies of lower limbs can be asymmetric at the onset of independent walking and improve symmetry with age, whereas muscle synergies do not show an age-related effect on symmetry. The current results can be used as data for normative motor synergy symmetry. A future study comparing children with motor disorders will be conducted.

## Methods

### Participants

The study included 13 preschool children (G1: ages 2.0–5.9 years, with 4.25 ± 1.27 years), thirteen children (G2: ages 6.0–9.9 years, with 7.46 ± 1.00 years), and thirteen pubertal children (G3: ages 10.0–13.9 years, with 12.22 ± 1.43 years). Table 3 shows their demographic and anthropometric characteristics. There were no neurological diseases or previous lower limb pathology or surgery in any of the individuals. Before participating in the protocol, all participants signed informed consent or parental consent. The study was approved by the ethics committee of the children’s hospital of Chongqing medical university.

**Table 3:** Participants' demographic and anthropometric characteristics

	Preschool children (2.0–5.9 years)	Children (6.0–9.9 years)	Pubertal children (10.0–13.9 years)
Participants # (M, F)	13 (7 M, 6F)	13 (6 M, 7F)	13 (7 M, 6F)
Age (years)	4.25 ± 1.27	7.46 ± 1.00	12.22 ± 1.43
Height (cm)	99.53 ± 11.87	119.07 ± 6.96	150.07 ± 6.98
Weight (kg)	16.92 ± 3.86	23.76 ± 3.26	39.76 ± 5.43
Body mass index (kg m^−2^)	16.92 ± 1.03	16.76 ± 0.59	17.46 ± 0.96

Values are expressed as mean ± SD

### Data acquisition

**Fig. 4:**
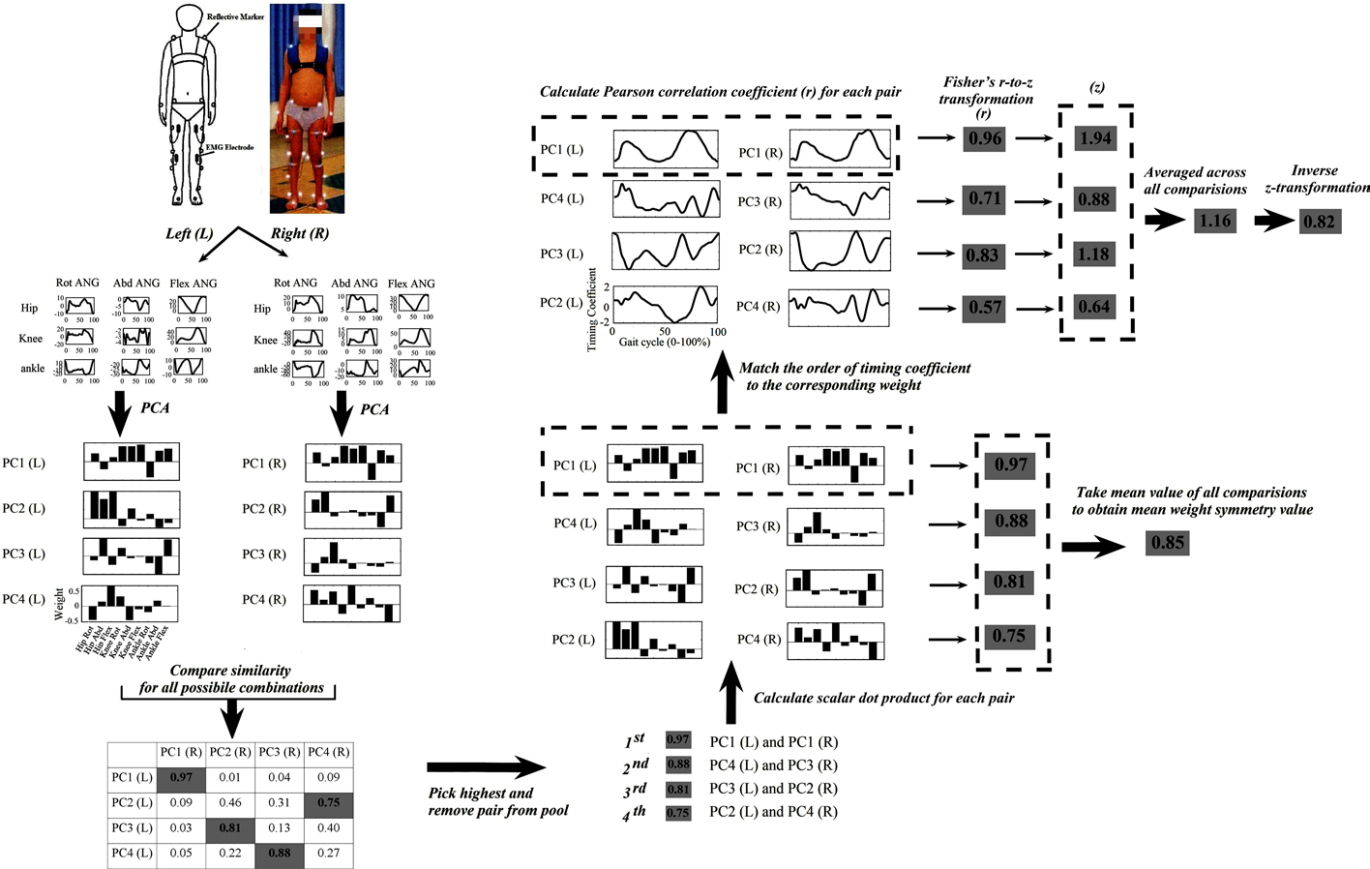
Schematic overview of the calculation steps for the mean symmetry values of kinematic synergies. Step 1: participants underwent a 3DGA model with total-body kinematics using a motion capture system. Step 2: from the 3DGA model, the joint angles of the hip, knee, and ankle were calculated concerning the flexion–extension (Flex), abduction–adduction (Abd), and internal–external rotation (Rot). In this example, the angle of the three lower joints is presented during one gait cycle. The joint angles involving the 9 DoFs were pooled together as an original joint matrix. Step 3: PCA was employed to decompose the original joint matrix into two components, synergy weight, and timing coefficient. Step 4: the similarities of all possible combinations of the synergy weight on the left to the right side were calculated with the scalar dot product [23]. The pair with the highest value was removed from the pool and the process continues until all kinematic synergies were matched. Step 5: the weight of synergies on both sides of each pair was compared with the scalar dot product [69, 70], while the symmetry value of all comparisons was averaged as the mean weight symmetry value. Step 6: the similarity of the corresponding timing coefficients for each pair was evaluated with the Pearson correlation coefficient (*r*). These correlation values were normalized using Fisher’s *r*-to-*z* transformation and then averaged across all comparisons. Inverse *z*-transformation was finally performed to transform the averaged *z* value back to *r*, which was considered as the mean timing symmetry value

Six high-speed digital cameras were used to record kinematic data of the participants at 60 frames per second using a motion capture system (Raptor-E, Motion Analysis Inc., Santa Rosa, CA, USA). EMGs were recorded using the MA-300 EMG system (Motion Lab Systems, Inc., USA) at a sampling rate of 2000 Hz. According to Helen Hayes’s marker set, the shoulder (lateral to the acromion), elbow (lateral epicondyle), wrist (ulnar styloid process), hip (posterior superior iliac spine), knee (lateral joint line), ankle (lateral malleolus), and trunk (shoulder blade) et al., were all taped with reflective markers, as shown in Fig. 4. Surface EMG was recorded from the Vastus Medialis (VM), hamstrings [Semitendinosus] (HAM), tibialis anterior (TA), Gastrocnemius [Medial Head] (GAS), and gluteus maximus (Gmax) as well. EMG recording and marker tracking were all synchronized.

For multiple trials, participants walked barefoot at their own pace along a 7-m path. We were able to determine individual strides with full marker visibility. A successful sequence of alternate steps based on the existence of at least three complete, consecutive strides was defined as a valid trial. Ten trials from each participant were analyzed. For data analysis, only the walking sequences in which the participant walked straight without stopping or diverting were chosen. To reduce the influence of walking acceleration or deceleration, the first and last steps of each sequence (beginning and stopping) were also omitted from the data analysis.

### Data analysis

#### Data pre-processing

The raw kinematic data were smoothed using a Butterworth 2nd order low-pass filter (10 Hz cut-off frequency) before being used to calculate joint angles. Joint angles of bilateral lower extremities were calculated using OrthoTrak kinematic analysis software (Motion Analysis, Santa Rosa, CA, USA). The joint angles for each gait stride were then segmented depending on the time it takes for the heel strike. A heel strike is determined to be the point where the elevation of the heel reflective marker is at the lowest point. All strides were interpolated as a percentage of gait stride time (0 percent–100 percent) due to differences in stride duration between trials and subjects. Each participant's normalized trials for each joint were averaged across trials. Then, the averaged joint angles were normalized to have zero mean and unit variance.

#### Kinematic synergies extraction

The multi-joint synergy model that was created for each lower limb during walking had 9 DoFs including hip (3 DoFs: flexion–extension (Hip-Flex), abduction–adduction (Hip-Abd), and internal–external rotation (Hip-Rot)), knee (3 DoFs: flexion–extension (Knee-Flex), abduction–adduction (Knee-Abd), and internal–external rotation (Knee-Rot)), ankle (3 DoFs: flexion–extension (Ankle-Flex), abduction–adduction (Ankle-Abd), and internal–external rotation (Ankle-Rot)). The joint angles involving the 9 DoFs were pooled together as an original joint motion matrix (${M}^{m\times t}$) for each participant to extract multiple-joint synergies for each lower limb. The original joint motion matrix was decomposed into two components, synergy weight (*W*, or synergy structure), and timing coefficient (*C*, or relative time-varying activation of those synergies), as described by the following equation:

$${M}^{m\times t}\cong {W}^{m\times n}{C}^{n\times t},$$

where *n* is the number of principal components (PCs), also known as synergies, and *m* is the number of joint DoFs (in this study, *m* = 9). *C*, also known as the timing coefficient, is an $n\times t$ matrix with t being the number of time points (101 in this study across the normalized gait cycle). Synergies are defined by the number of PCs. The first PCs had the most variance explained, the second PCs had the second-highest variance explained, and so on (as shown in Table 1) [71]. According to the criterion established by prior studies, the number of PCs (i.e., synergies) was chosen as a plausible definition of synergies when it explained more than 90% of the variance [35, 72].

#### Muscle synergies extraction

To eliminate power interference, the sEMG signals were band-pass filtered using a fourth-order, zero-phase Butterworth digital filter with a frequency band of 10 to 400 Hz and a 50 Hz digital notch filter. The EMG data were segmented for each gait stride according to the initiation of heel contact after the noise was removed. To create the linear envelope, the pre-processed EMG signals were first demeaned, rectified, and then low-pass filtered with a zero lag fourth-order low-pass (9 Hz) [73, 74]. Each muscle's envelope was then normalized to its peak value among all trials, resampled from 0–100 percent of the walking stride at the 1% step increase [75], and finally averaged across all strides performed by an individual subject, giving a matrix of 5 (five muscles) by 101 (0–100% gait stride) for each limb.

Since the nonnegativity of non-negative matrix factorization (NNMF) satisfies the physiological significance for muscle activations, NNMF was used to extract muscle synergies from EMG data [23, 24, 33]. The following equation explains how this method divided the measured EMG data matrices (*V*) into two components, spatial structure (S, termed the weight of synergies) and timing coefficient (*H*, or relative time-varying activation of those synergies):

$${ V}^{m\times t}\cong {S}^{m\times n}{H}^{n\times t}.$$

In this equation, S is an $m\times n$ matrix where m is the number of muscles (in this study *m* = 5) and *n* is the number of muscle synergies. *H* is an $n\times t$ matrix where *t* is a number of time points (101 across the normalized gait cycle in this study). Thus, each column of S represents the relative weight of muscles in each synergy and each row of H represents the activation level of each synergy over the gait cycle. NNMF was repeated within an iterative optimization, which minimized the sum of squared error between the activations calculated by *S* × *H* and the measured EMG data matrices (*V* )[76].

**Fig. 5:**
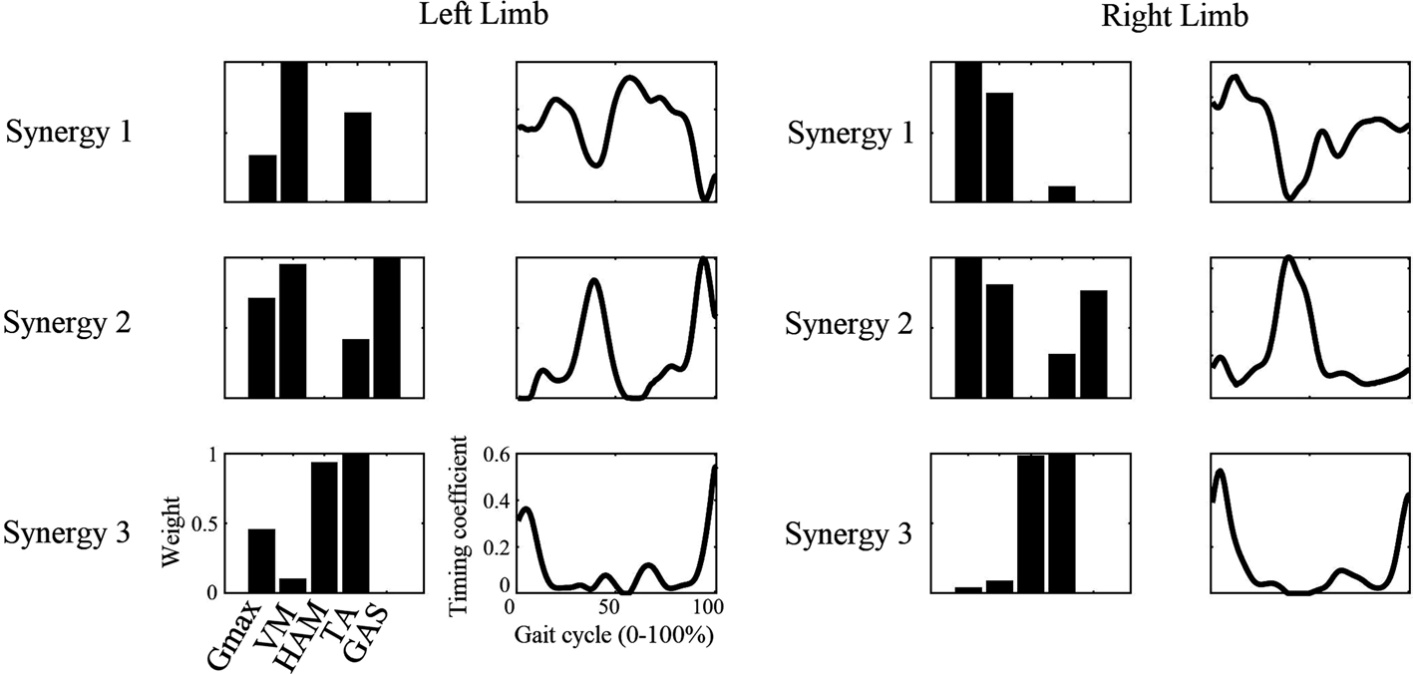
Representative subject with all muscle synergies identified by non-negative matrix factorization (NMF) algorithm

With regard to the number of muscle synergies, we did not make any presumptions about how many muscle synergies would be required to properly reconstruct the original EMG data matrices (*V*). The variance accounted for (VAF, ranging from 0 to 1), which is defined as $\mathrm{VAF}=1-{\Vert V-S\times H\Vert }^{2}/{\Vert V\Vert }^{2}$ [23, 24, 34], was used to measure the goodness of fit of the data reconstruction. For each subject, we identified the least number of muscle synergies that satisfied the following two criteria: (1) the total reconstructed EMGs counted for at least 90% of the variance across all muscles (VAF > 90%); (2) each reconstructed EMGs counted for greater than 75% VAF of the measurement from the corresponding single muscle. These criteria are thought to be conservative in order to guarantee the accuracy of the reconstruction [24]. A typical decomposition result is shown in Fig. 5.

#### Symmetry calculation based on the kinematic and muscle synergies

It should be noted that direct comparison is possible by imposing the same number of kinematic synergies (or muscle synergies) on the left and right sides. Taking the kinematic synergies as an example, the number of kinematic synergies was determined based on a variance threshold value of 90% [72]. However, this could lead to the left and right sides may have a different number of synergies, which makes a direct comparison between the left and right sides difficult. Hence, in the current study, we choose the maximum number of synergies on the left and the right as the number of synergies used for symmetry comparison. For example, if three synergies were identified for the left and four synergies were identified for the right side according to the variance threshold, then the number of synergies was chosen to four for both sides. Then, the comparison of the weight of kinematic synergies on the left to the right side was calculated with the scalar dot product. The pair with the highest value was taken out of the pool, and the process was repeated until all kinematic synergies were matched. This matching process was repeated for all subjects.

After the matching process, synergy weights (${W}^{m\times n}$) and the corresponding timing coefficients (${C}^{n\times t}$) of left and right side were compared to obtain a value to denote the symmetry between them. These values will be referred to as the “weight symmetry” (for synergy weight symmetry) and “timing symmetry” (for timing coefficient symmetry). The weight of synergies for each pair was compared with the scalar dot product, while the symmetry value of all comparisons was averaged as the mean weight symmetry value.

Additionally, the similarity of the corresponding timing coefficients for each pair was evaluated with the Pearson correlation coefficient (*r*). In order to normalize intra-individual variance, the absolute individual Pearson correlation coefficient (*r*) value was Fisher z-transformed to get a value (z), using the following equation:

$$z=\mathit{ln}((1+r)/(1-r))/2.$$

The *z*-transformed values (*z*) of all comparisons were averaged and then inverse *z*-transformation was performed to transform the averaged *z* value back to *r* (as shown in the following equation), which was considered as the mean timing symmetry value:

$$r=\frac{{e}^{(2z)}-1}{{e}^{(2z)}+1}.$$

An infographic of the synergies matching and symmetry calculation procedure is provided in Fig. 4.

### Statistical analysis

A one-way analysis of variance (ANOVA) was used to analyze the differences in symmetry values of kinematic synergies and muscle synergies introduced by age group (G1, G2, G3). The age range of the participants (e.g., G1, G2, G3) was the independent variable, and the mean symmetry value determined from kinematic synergies and muscle synergies was the dependent variable. If there was a significant effect, a Bonferroni post hoc test was used. Furthermore, if there was a significant group difference, Spearman's rho rank order correlation was used to examine the relationship between age and gait symmetry values, with the level of significance set at *p* = 0.05. According to Cohen’s guidelines [77], the rho values of 0.1, 0.3, and 0.5 were considered representative of small, moderate, and large correlations, respectively. All analyses were carried out using the IBM SPSS software (IBM, Armonk, NY, USA).

## Data Availability

The datasets used and/or analyzed during the current study are available from the corresponding author upon reasonable request.
